# Intestinal Microfold Cells Play a Critical Role in the Uptake and Oral Tolerance Mediated by Lysophosphatidylserine-Containing Lipidic Nanoparticles

**DOI:** 10.3390/nano16070412

**Published:** 2026-03-29

**Authors:** Vincent Chak, Sujay Harne, Jason G. Kay, Elizabeth Wohlfert, Sathy V. Balu-Iyer

**Affiliations:** 1 Department of Pharmaceutical Sciences, School of Pharmacy and Pharmaceutical Sciences, University at Buffalo, The State University of New York, 359 Pharmacy Building, Buffalo, NY 14214, USA; vchak@buffalo.edu (V.C.); sujayhar@buffalo.edu (S.H.); 2 Department of Oral Biology, School of Dental Medicine, University at Buffalo, The State University of New York, Buffalo, NY 14214, USA; jasonkay@buffalo.edu; 3 Department of Microbiology and Immunology, Jacobs School of Medicine and Biomedical Sciences, University at Buffalo, The State University of New York, Buffalo, NY 14214, USA; wohlfert@buffalo.edu

**Keywords:** phosphatidylserine, nanoparticle, oral tolerance, apoptosis, regulatory T cells, immune regulation, intestinal-M-cells

## Abstract

Phosphatidylserine (PS) is an anionic phospholipid that is exposed to the outer leaflet of the cell membrane during apoptosis. This PS externalization can teach the immune system to tolerate an antigen without eliciting immunological consequences. Previously, we showed that mice treated with PS nanoparticles containing single-chain PS (LysoPS) induced oral tolerance towards therapeutic proteins, whereas double-chain PS did not. These observations suggest that structural alterations of PS play a critical role in its tolerogenic potential. Given that intestinal microfold cells (M-cells) facilitate the transport of particulate antigens from the intestinal lumen to Peyer’s patches (PP) for immune surveillance, we hypothesized that the failure of double-chain PS to induce tolerance may result from insufficient uptake by M-cells. The M cell-mediated uptake was investigated using in vitro and ex vivo studies and oral tolerance towards ovalbumin (OVA) was studied in M-cell-deficient mice. Consistent with this hypothesis, our data showed that LysoPS nanoparticles displayed at least a 2-fold increase in immune cell exposure and M-cell-mediated uptake compared to double-chain PS-containing nanoparticles. Importantly, LysoPS-mediated oral tolerance was absent in M cell-deficient mice with higher anti-ova antibody titers than the wild-type strain. These studies demonstrate that higher PS exposure on LysosPS nanoparticles compared to double chain could play a significant role in M cell-mediated tolerance.

## 1. Introduction

Phosphatidylserine (PS) is a phospholipid composed of two acyl chains linked to the serine headgroup through a glycerol backbone. Under homeostatic conditions, PS is predominantly located in the inner leaflet of the eukaryotic cell membrane [1]. When cells undergo apoptosis, PS flips from the inner leaflet to the outer leaflet of the cell membrane to engage with phagocytes. These PS-expressing apoptotic cells send “find-me”, “eat-me” and “ignore-me” signals to antigen-presenting cells (APCs), facilitating the removal of cell debris without further immunological consequence [2,3,4]. Therefore, we utilized the mechanism of immunological ignorance of PS to prevent immunogenicity of therapeutic proteins such as Factor VIII (FVIII) and acid alpha-glucosidase (GAA). Our studies showed that the immune system actively learned not to mount a response in an antigen-specific manner towards these therapeutic proteins, suggesting that PS promotes immunological tolerance rather than immunological ignorance [5,6]. Mice pre-treated with PS loaded with FVIII, administered subcutaneously or intravenously, successfully suppressed the development of anti-FVIII neutralizing antibody (nAb) titers following FVIII rechallenge, while rechallenge with non-cross-reactive antigen elicited a normal response in PS-FVIII pre-treated animals. Importantly, mice treated with FVIII in the presence of the general immune inhibitor dexamethasone (Dex) developed FVIII-specific nAb once treatment had stopped. The structure of PS also influences its immunoregulatory properties, with variations in acyl-chain number, length, and degree of unsaturation impacting its biophysical characteristics and tolerance potential. Mice treated subcutaneously with mono-acyl chain of PS, known as LysoPS, containing FVIII or acid alpha-glucosidase (GAA), also reduced antibody development [7,8], similar to a double-chain PS liposome. However, when the route of administration switched to oral, only mice treated with LysoPS induced oral tolerance, whereas those treated with double-chain PS nanoparticles did not [9]. This observation highlights the role of PS structure in oral tolerance.

Oral tolerance is a specialized immunological process in the mucosal immune system that naturally promotes immune tolerance to ingested antigens [10]. This biological process is evolutionarily important, allowing organisms to acquire dietary antigens while keeping pathogens away. Oral tolerance is primarily initiated in the gut-associated lymphoid tissues (GALTs), which serve as key sites for antigen sampling and as inductive sites for adaptive immune responses [11]. GALTs consist of three main components: Peyer’s patches (PPs), lamina propria (LP), and the mesenteric lymph node (MLN). The LP, located beneath the intestinal epithelium, is populated with diverse populations of immune cells, including dendritic cells (DCs), macrophages, T cells, and B cells, all of which collectively contribute to mucosal immune tolerance. Meanwhile, PPs are organized lymphoid aggregates distributed along the small intestine. They contain semi-permeable intestinal epithelial cells, known as intestinal microfold (M) cells, which play a crucial role in transporting luminal antigens, including proteins and vesicles of various sizes and shapes, to the underlying lymphoid follicles, where antigen sampling and immune priming occur [12,13]. In addition to transporting antigens, intestinal M-cells are reportedly involved in the regulation of immune responses. Studies have shown that intestinal M-cells exposed to a low dose of ovalbumin (OVA) conjugated with a ligand targeting intestinal M-cells enhanced oral tolerance [14,15], suggesting intestinal M-cells are actively involved in induction of oral tolerance.

In our previous studies, we demonstrated that nanoparticles constructed with double-chain PS nanoparticles failed to induce oral tolerance, whereas LysoPS nanoparticles successfully induced tolerance, even when the nanoparticles had comparable size and morphology [9]. These observations suggested that the structural features of PS may play a critical role in determining its tolerogenic potential; however, the mechanisms underlying this phenomenon remain poorly understood. Given that the structure of PS influences oral tolerance and that intestinal M-cells play an important role in antigen sampling for tolerance induction, we hypothesized that intestinal M-cells are critical for PS-mediated oral tolerance and that structural variations in PS would affect their interaction with intestinal M-cells and subsequent tolerance responses. To test this hypothesis, we investigated M cell uptake and oral tolerance induced by LysoPS and double chain PS containing OVA using in vitro, ex vivo and in vivo approaches. These studies demonstrate that M-cells play an important role in facilitating PS-mediated intestinal uptake and downstream tolerance induction.

## 2. Materials

1-oleoyl-2-hydroxy-sn-glycero-3-phospho-L-serine (sodium salt; 18:1 LysoPS, CAS number: 326589-90-6), L-α-phosphatidylserine (BrainPS, CAS number: 383907-32-2), and 1,2-dimyristoyl-sn-glycero-3-phosphocholine (DMPC; CAS number: 18194-24-6) were purchased from Avanti Polar Lipids, Inc. (Alabaster, AL, USA). DiI dye and fluorescein-conjugated ovalbumin (FITC/AF648) were purchased from Invitrogen (Thermo Fisher Scientific, Waltham, MA, USA). Endograde ovalbumin was purchased from BioVendor LLC (Asheville, NC, USA). All solvents, buffer salts, and micro-BCA kits were purchased from Fisher Scientific (Fairlawn, NJ, USA). The Endosafe Endochrome-K^®^ Kit was purchased from Charles River Laboratories (Charleston, SC, USA). Antibodies used for flow cytometry staining were obtained from eBioscience (Thermo Fisher Scientific, San Diego, CA, USA), BioLegend (San Diego, CA, USA), and Cytek Biosciences (Fremont, CA, USA). The multiplex TGF-β cytokine kit was purchased from Sigma-Aldrich (St. Louis, MO, USA).

### 2.1. SPF Conditional RANK Knockout Mice

B6.Cg-Tnfrsf11a^tm1.1Irw^/J stain (Strain #:027495), villin-cre mice (Tg(Vil-cre)997Gum/J strain (Strain #:004586) and background strain C57BL/6J (B6, Strain #:000664) were obtained from Jackson Laboratory (Bar Harbor, ME, USA). B6.Cg-Tnfrsf11a^tm1.1Irw^/J stain mice were cross-bred with villin-cre mice (Tg(Vil-cre)997Gum/J strain to generate homozygous floxed RANK mice carrying the villin-cre transgene (villin-cre RANKF/F genotype; also designated RANKΔIEC mice) [16]. These mouse colonies were generated in the Roswell Park Comprehensive Cancer Center (Buffalo, NY, USA). All mice were genotyped for the expression of both Vil-cre and Tnfrsf11a-3 to ensure that mice used in this study expressed the correct genotypes. This process was conducted by contract genotyping services (TransnetYX, Inc., Cordova, TN, USA). C57BL/6J and RANKΔIEC mice were housed in a specific pathogen-free (SPF) facility. Both male and female mice, 8 to 20 weeks old, were used in this study. All animal experiments were conducted in strict accordance with and with approval from the Institutional Animal Care and Use Committee (IACUC) at the University at Buffalo, the State University of New York.

### 2.2. Preparation of Nanoparticles

PS nanoparticles were prepared at a 30:70 molar ratio of PS to 14:0 DMPC using the thin-film dehydration method described previously [8]. Lipid films were rehydrated in 5 mM citrate buffer (pH 4.0) and extruded through 100 nm polycarbonate filter membranes multiple times using a high-pressure extruder. Final lipid concentration and recovery were determined by phosphate assay [17]. The protein-to-lipid molar ratio was fixed at 1:1000 throughout the study. Ovalbumin (OVA) was incorporated by incubating protein with nanoparticles at 37 °C for 30 min. To ensure nanoparticles were uniformly sized at 100 nm, nanoparticle diameters were measured using a Nicomp 380 Submicron Particle Sizer (Entegris, Santa Barbara, CA, USA) with intensity-weighted Gaussian analysis. Endotoxin levels were monitored using the Endosafe Endochrome-K endotoxin assay kit (Charles River Laboratories, Charleston, SC, USA), and only formulations with endotoxin levels < 0.05 EU/mL were used for the in vivo studies.

### 2.3. In Vitro M Cell Uptake Study

The impact of PS structure on nanoparticle transport was assessed using an in vitro intestinal M cell co-culture model, following a protocol adapted from the literature [18]. Briefly, Caco-2 cells were seeded on transwell inserts and co-cultured with human Raji B cells to induce differentiation into an M cell-like monolayer. PS nanoparticles containing OVA were added to the apical compartment at 0.208 μmol/mL in complete medium (DMEM with 10% FBS and 1% penicillin-streptomycin), while 1 mL of complete medium was added to the basolateral compartment. Cultures were incubated at 37 °C and sampled at 0, 0.25, 0.5, 1, 2, and 3 h. To assess nanoparticle transport, transepithelial electrical resistance (TEER) was measured using an epithelial voltohmmeter to evaluate membrane integrity and permeability of the monolayer. TEER is a sensitive method that detects changes in tight junction integrity, with reductions in TEER indicating increased permeability and potential nanoparticle translocation. In this study, TEER values were expressed as fold-changes relative to the 0 h baseline. All measurements were corrected for blank media resistance, and the average fold-change from each group was calculated from five independent biological samples.

### 2.4. PS Nanoparticle Exposure in the Intestinal M-Cells and MLN

The impact of PS structure on intestinal M cell uptake was evaluated using a ligated gut-loop assay in gender-mixed Swiss Webster mice (20–40 g), as previously described [19]. Mice were anesthetized with 3% isoflurane and placed on a heat pad before undergoing a non-survival surgical procedure. A small abdominal incision was made to expose the intestine, and Peyer’s patches (PPs) located near the ileum were identified. Approximately 1 cm of intestine containing a PP was ligated at both ends using silk sutures. A 100 μL volume of the samples (double-chains PS or LysoPS containing 20 μg of OVA-AF488), was injected into the isolated intestinal loop using a 31G prefilled syringe, and the loop was incubated in situ for one hour. Following the incubation, the ligated gut loops were excised, rinsed with cold PBS, and processed into single-cell suspensions. Tissues were first incubated with pre-digestion buffer (phenol red-free RPMI 1640 supplemented with 200 μg/mL dithiothreitol, 25 mM HEPES, and 2% fetal bovine serum) for 20 min, followed by enzymatic digestion with collagenase D and DNase I in phenol red-free RPMI 1640 for an additional 20 min. Resulting single-cell suspensions were stained with fluorescent antibodies specific for immune cell markers. Flow cytometry counting beads were added to samples prior to data acquisition to enable quantification of absolute cell numbers and nanoparticle-ova positive immune cell populations. Samples were subsequently analyzed by flow cytometry. To assess PS nanoparticle exposure in the MLN, mice were orally administered fluorescently labeled nanoparticles containing OVA after six hours of fasting, and MLNs were collected three hours post-exposure. MLNs were homogenized into single-cell suspensions, and nanoparticle exposure was assessed using flow cytometry. Single-cell suspensions from both PPs and MLNs were stained for B cells (B220), T cells (CD3), dendritic cells (CD11c), and macrophages (F4/80) to assess nanoparticle uptake by immune cells.

### 2.5. Oral Tolerance Study

Mixed gender of C57BL/6J (n = 8/group) and RANKΔIEC (n = 7/group) mice were prophylactically treated with buffer, 1 μg of OVA protein, or 1 μg of OVA protein in the presence of LysoPS nanoparticles, once weekly for 9 weeks. Starting at week 6, mice were rechallenged weekly with 2 μg of free OVA subcutaneously, 24 h after oral administration, for four weeks. Mice underwent a two-week washout period after the final rechallenge. All mice were sacrificed, and plasma was collected via cardiac puncture containing 10% *v*/*v* acid citrate dextrose (ACD). Plasma, spleen, and bone marrow were collected for OVA-specific IgG titer measurement and immunophenotyping of T cells and B cells.

### 2.6. Anti-OVA IgG Antibody ELISA

Anti-OVA IgG ELISA kits were purchased from Chondrex (Woodinville, WA, USA) and assays were conducted following the manufacturer’s protocol.

### 2.7. Flow Cytometry

Single-cell suspensions were prepared from tissues and stained with the following reagents: LIVE/DEAD™ Fixable Blue Dead Cell Stain Kit (Cat# L34962, Life Technologies), CD138 BUV395 clone 281-2 (Cat# 740240), CD49b BV750 clone HMα2 (Cat# 746974), CD223 BUV563 clone C9B7W (Cat# 741350), Foxp3 R718 clone 3G3 (Cat# 567465), APC/Fire™ 810 anti-mouse CD4 (Cat# 100480), Spark YG™ 570 anti-mouse/human CD45R/B220 (Cat# 103286), anti-mouse TGF-β1 PerCP-Cy5.5 (Cat# 141410), and cFluor^®^ R659 anti-mouse CD25 (Cat# R7-20575). Intracellular staining was performed after fixation and permeabilization steps. Fluorescence Minus One (FMO) control was performed for gating strategies. Flow cytometric data were acquired on a Cytek^®^ Aurora 5-laser spectral flow cytometer (UV/V/B/YG/R; 64 + 3 channels; Fremont, CA, USA) and analyzed using FlowJo™ software version 10 (Ashland, OR, USA).

### 2.8. Multiplex TGF-Beta Cytokines Assay

Plasma samples collected in week 7, 12 h after OVA rechallenge via the subcutaneous route, were analyzed for TGF-β1 concentration using the MILLIPLEX TGFBMAG-64K-03 kit (Sigma-Aldrich, St. Louis, MO, USA). Following the manufacturer’s protocol, samples were diluted at a ratio of 1:2 or 1:3, depending on plasma volume, and assays were performed at the Roswell Park Comprehensive Cancer Center.

### 2.9. Statistical Analysis

All statistical analyses were conducted using GraphPad Prism version 10.3 (GraphPad Software, La Jolla, CA, USA). One-way or two-way ANOVA followed by Dunnett’s post hoc analysis on the original or log-transformed values was performed to detect significant differences (*p* < 0.05) as indicated. For comparisons between two groups, unpaired two-tailed Student’s *t*-tests were performed between animal models. Unless otherwise stated, all data are presented as mean ± standard error of the mean (SEM).

## 3. Results

### 3.1. LysoPS Nanoparticles Enhance Intestinal M-Cells Absorption for Oral Tolerance

**Figure 1:**
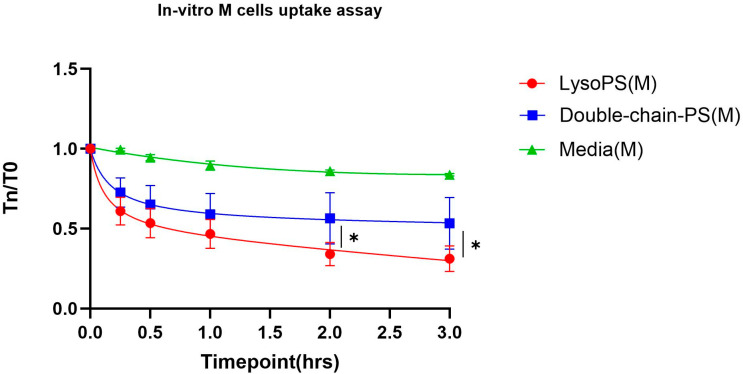
PS nanoparticle packing defects altered intestinal M-cells uptake in vitro. An in vitro intestinal M cell co-culture model was used to assess nanoparticle uptake between double-chain PS and LysoPS nanoparticles using transepithelial electrical resistance (TEER) measurements. The red circle line represents LysoPS nanoparticles, the blue square line represents double-chain PS nanoparticles, and the green triangle line represents media over time. TEER was measured from 0 to 3 h, and data are presented as the TEER value at each specified time point (Tn) normalized to the initial TEER value at T = 0 (T0). Results are plotted as the average of 3 independent biological samples. Statistical significance was evaluated using two-way analysis of variance (ANOVA) with Dunnett’s multiple comparison test. * *p* < 0.05.

The impact of PS structure on nanoparticle transport via intestinal M-Cells was evaluated using both in vitro and ex vivo experiments. For the in vitro study, we used a well-established human M cell co-culture model [18]. This model was generated by co-culturing Caco-2 epithelial cells with Raji B cells, a method that induces the differentiation of Caco-2 cells into M cell-like specialized epithelial cells. These induced M-cells recapitulate several key features of native intestinal M-cells, including cell morphology, reduced density of microvilli, and enhanced expression of M cell-associated markers such as Glycoprotein 2 (GP2) and Spi-B [20]. By using this M cell-like model, we evaluated the impact of PS structure on intestinal M-cells uptake using Transepithelial electrical resistance (TEER). TEER measures the ionic conductance across the cell layer: high TEER values indicate intact tight junctions and low paracellular permeability, while a decrease in TEER indicates disruption of tight junctions or increased transcellular or paracellular transport. Therefore, changes in TEER measurement indicate the degree of epithelial barrier opening and potential for nanoparticle translocation. Our data showed that after 2–3 h incubation, LysoPS-OVA treatment resulted in a significantly greater reduction in TEER relative to double-chain PS-OVA. Specifically, LysoPS-OVA induced a sustained and progressive decrease in resistance, whereas the TEER values in the double-chain PS group plateaued (Figure 1). These results suggest that LysoPS-OVA nanoparticles enhanced nanoparticle trafficking through M-cells over time.

**Figure 2:**
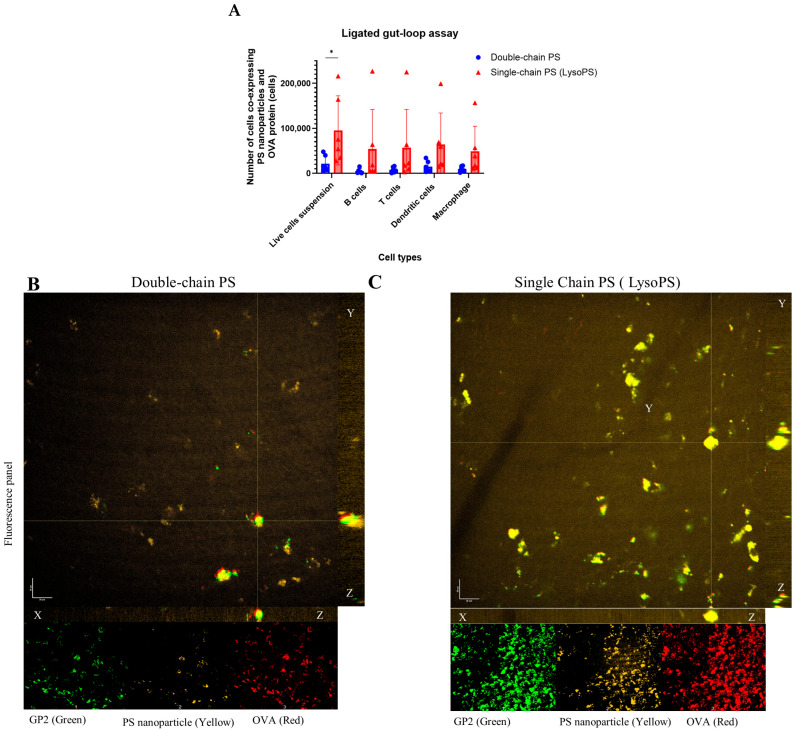
PS nanoparticle altered intestinal M-cells uptake in vivo. PS nanoparticle engagement with intestinal M-cells assessed using an intestinal ligated gut loop assay. Peyer’s patches (PPs), which contain intestinal M-cells, were used for this assay. The PP located closest to the ileum was selected for the ligated gut loop experiment. (**A**) Bar chart showing the number of live cells co-expressing fluorescently labeled PS nanoparticles and OVA protein, with distribution among. Result is plotted as average of 6 independent biological samples. Error bars represent mean ± SD. Statistical analysis was performed using multiple unpaired *t*-tests without correction for multiple comparisons; significance is indicated as * *p* < 0.05. (**B**,**C**) Representative confocal fluorescence images of whole PP tissue comparing double-chain PS and LysoPS. Images include an XY panel, a YZ panel (right), and an XZ panel (below the XY panel). Individual fluorescent channels are also shown at the bottom. The images display fluorescence intensity for GP2-expressing intestinal M-cells (green), Dil-labeled PS nanoparticles (yellow), and Alexa Fluor 648-labeled OVA protein (red). Scale bar = 20 µm.

To validate this observation, an additional intestinal M cell transport experiment was performed using an ex vivo intestinal gut-loop assay. This experiment mimics the physiological environment of intestinal M-cells and allows the study of cellular transport. Both PS formulations containing OVA were directly injected into ligated gut loops-intestinal segments containing Peyer’s patches (PPs)-to assess nanoparticle uptake by immune cells, including B cells, T cells, macrophages, and dendritic cells (DCs), using flow cytometry and confocal fluorescence microscopy. The results showed that PPs treated with LysoPS-OVA had a significantly higher frequency of cells co-expressing OVA fluorescence and PS fluorescence than those treated with double-chain PS-OVA (Figure 2A). In addition, our data also identified that B cells, T cells, DCs, and macrophages all displayed higher uptake of LysoPS-OVA compared to double-chain PS-OVA, suggesting higher interactions of LysoPS-OVA with these immune cells, which may contribute to immune tolerance Furthermore, nanoparticle uptake by intestinal M-cells was visualized using fluorescence confocal imaging. Images showed that the number and fluorescence intensity of cells co-expressing PS-OVA were markedly higher in the LysoPS-OVA compared to double-chain PS-OVA (Figure 2B,C and Supplementary Figure S1A,B), supporting our observation from in vitro and the ligated gut loop assays that intestinal M-cells mediated uptake is higher in LysoPS nanoparticles compared to double-chain PS (Figure 2A).

Once antigens pass through intestinal M-cells, they interact with APCs in the PPs within the M cell pocket or in the subepithelial dome [21,22]. Eventually, antigens are captured by CD103+ expressing DCs [23], which upregulate C–C chemokine receptor type 7 (CCR7) expression and migrate to the mesenteric lymph node (MLN) for presentation to T cells or B cells [22]. Therefore, LysoPS-mediated oral tolerance could undergo a similar antigen presentation process to induce oral tolerance. In this study, we assessed the exposure of PS nanoparticles containing OVA in the MLN using flow cytometry. Mice receiving LysoPS-OVA have a significantly higher uptake in both live cells and DCs population compared to double-chain PS-OVA in the MLN (Supplementary Figure S2). This data supported that CCR7-expressing DCs are responsible for the transportation of antigens from PPs to MLN. It also suggests that DCs carried more fragments of LysoPS-OVA compared to double-chain PS-OVA.

### 3.2. LysoPS-OVA Induced OVA Specific IgG Antibody in RANKΔIEC Mice

**Figure 3:**
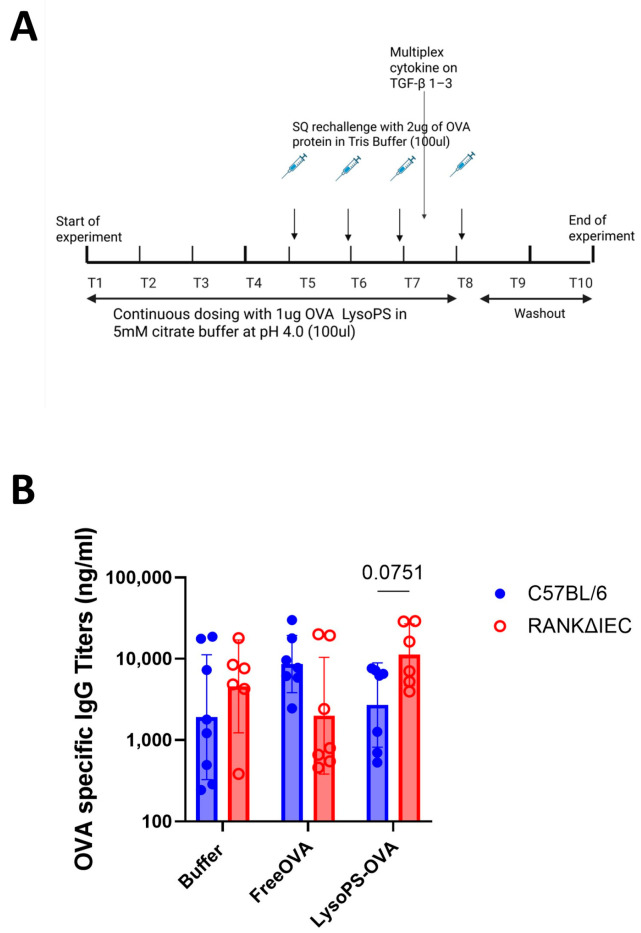
Absence of intestinal M-cells reverses LysoPS-mediated OVA-specific IgG antibody suppression. (**A**) The study design and timeline to investigate the role of intestine in LysoPS-mediated oral tolerance. (**B**) OVA-specific Immunoglobulin G (IgG) antibody titers across treatment groups C57BL/6 mice and RANKΔIEC mice. Result is plotted as average of 8 independent biological samples in C57BL/6 mice in each group and 6–7 independent biological samples in RANKΔIEC mice. Statistical significance was assessed using two-way ANOVA to compare differences between C57BL/6 and RANKΔIEC with Bonferroni multiple comparison.

An oral tolerance study was conducted in both C57BL/6 (background strain) and intestinal M cell knockout mice (RANKΔIEC), as described in the Methods section and Figure 3A. OVA-specific IgG titers between treatments were monitored and compared between animal models. Our data showed that C57BL/6 mice that received LysoPS-OVA had a trend of lower OVA-specific IgG antibody titers compared to animals that received Free OVA and buffer, while mice that received Free OVA generated higher anti-OVA IgG1 antibody titers within the treatment groups (Figure 3B). This observation is consistent with the literature reports that mice receiving low-dose antigen via oral administration increased serum allergen-specific IgG1 levels [24,25,26]. In addition, our previous studies also demonstrated that LysoPS treatment significantly reduced the development of anti-OVA antibody titers in the Swiss Webster mice under the same experimental conditions, supporting that LysoPS moderated antibody responses [27]). In contrast, our data showed that RANKΔIEC mice that received LysoPS-OVA had a trend of higher OVA-specific IgG antibody titers compared to animals that received Free OVA and buffer To evaluate the role of intestinal M-cells in regulating antibody responses induced by LysoPS-OVA, OVA-specific IgG titers were compared between two animal models receiving the same treatment. Our data showed that RANKΔIEC mice generated higher OVA-specific IgG antibody titers than C57BL/6 mice following LysoPS-OVA Notably, this difference was not observed in animals treated with buffer or Free OVA, suggesting that LysoPS-mediated oral tolerance was disrupted in the absence of intestinal M-cells.

### 3.3. LysoPS Suppressed Plasma Cell Differentiation

**Figure 4:**
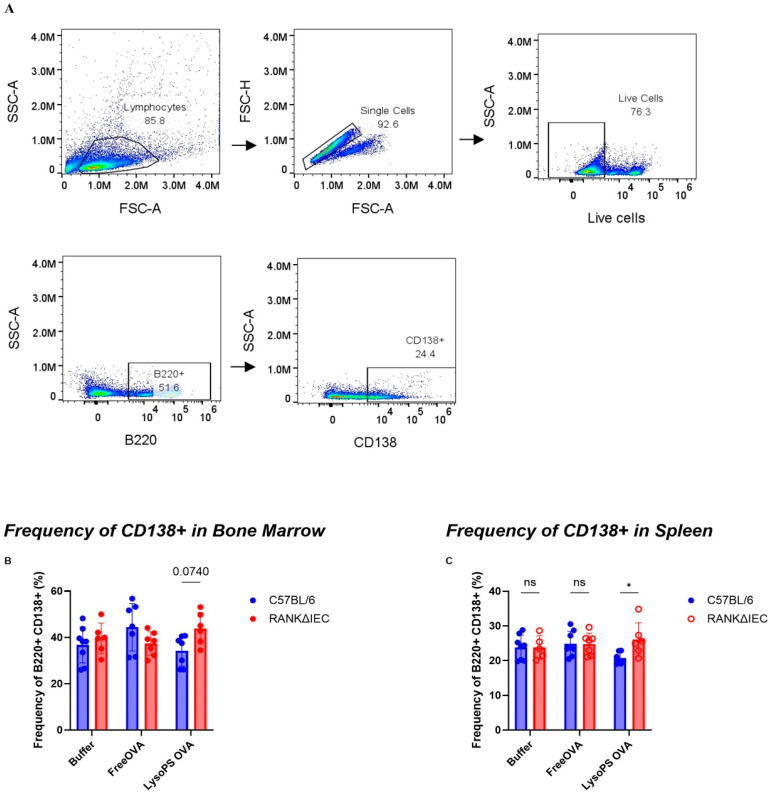
LysoPS reduced plasma cell formation in spleen and bone marrow. (**A**) Gating strategies for the frequency of CD138-expressing B cells in the spleen and bone marrow. Plasma cells were gated from the frequency of CD138 expression out of the B220+ B cells. (**B**) The frequency of plasma cells across treatment groups in C57BL/6 mice and RANKΔIEC mice in the bone marrow. (**C**) The frequency of plasma cells across treatment groups in C57BL/6 mice and RANKΔIEC mice in the Spleen. Result is plotted as average of 8 independent biological samples in C57BL/6 mice in each group and 6–7 independent biological samples in RANKΔIEC mice. Statistical significance was assessed using two-way ANOVA to compare differences between C57BL/6 and RANKΔIEC with Bonferroni multiple comparison. * *p* < 0.05; ns, not significant.

OVA-specific antibodies are secreted by plasma cells that differentiate from B cells. Therefore, the frequency of plasma cells is expected to be associated with the level of OVA-specific antibody titers. Using the gating strategy shown in Figure 4A, plasma cells were identified by CD138 expression within the B220^+^ population. In bone marrow, our data showed that LysoPS-OVA resulted in the lowest frequency of plasma cells compared to Free OVA or buffer in C57BL/6 mice (Figure 4B). This observation also supports our previous observation that LysoPS-OVA treatment significantly suppressed the frequency of plasma cells in Swiss Webster mice under the same experimental conditions, confirming that LysoPS moderated B cell responses [27]. In contrast, mice treated with LysoPS-OVA had a trend of higher frequency of plasma cells compared to buffer or Free OVA treatment. To assess the importance of intestinal M-cells in regulating plasma cell differentiation in response to LysoPS-treatment, we directly compared plasma cell frequencies between the two mouse models. Our data showed that C57BL/6 mice that received LysoPS-OVA significantly reduced frequency of plasma cells in the bone marrow compared to RANKΔIEC mice. Meanwhile, mice treated with buffer or Free OVA displayed similar plasma cell frequencies between the two models Similarly, in the spleen, our data showed that LysoPS-OVA resulted in the lowest frequency of plasma cells compared to Free OVA or buffer in C57BL/6 mice (Figure 4C) and a trend of higher frequency of plasma cells in the RANKΔIEC mice. We also found that LysoPS-OVA significantly reduced the frequency of plasma cells in the spleen compared to RANKΔIEC mice. These findings support our earlier observation that LysoPS-OVA suppresses plasma cell differentiation (both short-lived and long-lived plasma cells), thereby reducing the production of OVA-specific IgG antibodies. In contrast, the absence of intestinal M-cells abrogated LysoPS-mediated tolerogenic effects, resulting in B cell activation and plasma cell differentiation in both the spleen and bone marrow, leading to elevated OVA-specific IgG antibody titers.

### 3.4. LysoPS Induced Immunosuppressive Cytokine—Transforming Growth Factor-Beta (TGF-β)

**Figure 5:**
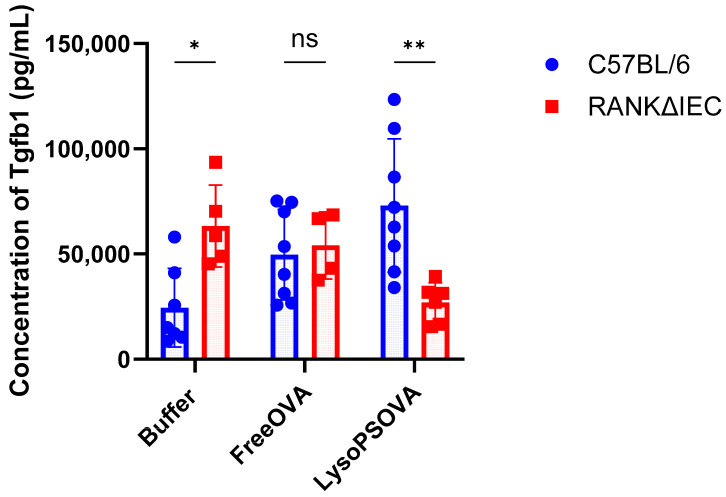
LysoPS induced transforming growth factor-beta (TGF-β) into plasma. Concentration of Transforming Growth Factor Beta isoform 1 (TGF-β1) measured using the MILLIPLEX TGF-β Magnetic Bead 3-Plex Kit- Immunology Multiplex Assay. Result is plotted as average of 8 independent biological samples in C57BL/6 mice in each group and 5–6 independent biological samples in RANKΔIEC mice. Statistical significance was assessed using two-way ANOVA to compare differences between C57BL/6 and RANKΔIEC with Bonferroni multiple comparison. * *p* < 0.05; ** *p* < 0.01; ns, not significant.

TGF-β is an immunosuppressive cytokine that plays a critical role in maintaining immune homeostasis and the generation of Tregs. Given its immunoregulatory function, it is possible that TGF-β contributes to LysoPS-mediated oral tolerance, and that the absence of intestinal M-cells may impair this pathway by reducing TGF-β production. To investigate the role of TGF-β in LysoPS-mediated oral tolerance, plasma samples were collected at week 7, 12 h after subcutaneous OVA rechallenge, in both animal models. Our data showed that C57BL/6 mice treated with LysoPS-OVA significantly increased plasma TGF-β1 levels compared with buffer (Figure 5). In contrast, this observation was reversed in RANKΔIEC mice, where mice treated with LysoPS-OVA significantly reduced the plasma TGF-β1 levels compared to buffer or Free OVA. To evaluate the roles of intestinal M-cells on plasma TGF-β1 levels, we directly compared the concentration of TGF-β1 across the two animal models in all treatment groups. We found that LysoPS-OVA treatment significantly lowered TGF-β1 production in RANKΔIEC mice compared to C57BL/6 mice, suggesting that the absence of intestinal M-cells impairs TGF-β1 induction in response to LysoPS-OVA, but not in Free OVA treatment. Interestingly, RANKΔIEC mice had significantly higher baseline TGF-β1 levels than C57BL/6 mice in the buffer-treated group, which may reflect a compensatory regulatory mechanism associated with the conditional knockout of RANK signaling in intestinal epithelial cells.

### 3.5. LysoPS-OVA Induced Regulatory T Cells (Tregs)

**Figure 6:**
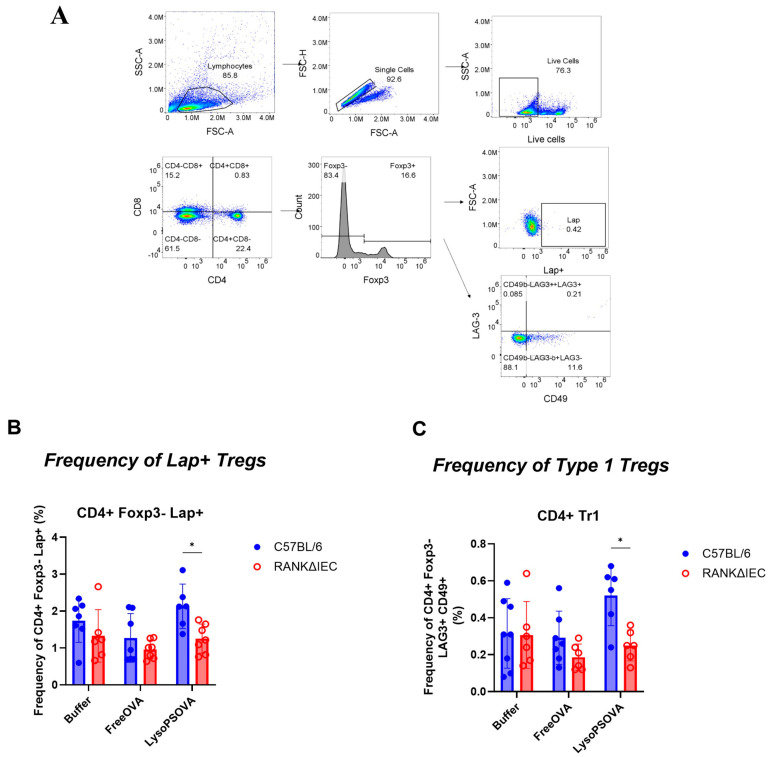
LysoPS induced Lap-expressing T cells in the spleen. Frequency of CD4+ Tregs after LysoPS treatment. (**A**) Gating strategies for CD4+ Lap+ T cells and Type 1 regulatory T cells (Tr1). Lap+ Tregs was gated from the frequency of Lap expression out of the CD4 + Foxp3- T cells. (**B**) The frequency of CD4+ Foxp3- Lap+ T cells across treatment group in C57BL/6 mice and RANKΔIEC mice in the spleen. (**C**) The frequency of CD4+ Tr1 T cells across treatment group in C57BL/6 mice and RANKΔIEC mice in the spleen. Result is plotted as average of 8 independent biological samples in C57BL/6 mice in each group and 6–7 independent biological samples in RANKΔIEC mice. Statistical significance was assessed using two-way ANOVA to compare differences between C57BL/6 and RANKΔIEC with Bonferroni multiple comparison.* *p* < 0.05.

Regulatory T cells play an important role in maintaining immune homeostasis and suppressing overactive immune responses. To investigate the role of regulatory T cells in LysoPS-mediated oral tolerance, we assessed the frequency of various Tregs at the end of the study. Spleens were collected and stained for CD4^+^ Foxp3^+^ CD25^+^ (Foxp3^+^ Treg), CD4^+^ Foxp3^−^ LAP^+^ (Th3 Treg), and CD4^+^ Foxp3^−^ CD49b^+^ Lag3^+^ (Tr1 Treg), using the gating strategies shown in Figure 6A and Supplementary Figure S4A. Our data showed that C57BL/6 mice treated with LysoPS significantly increased the frequency of Th3 Treg compared to Free OVA treatment (Figure 6B). However, the frequency of Th3Tregs was comparable across treatment groups in RANKΔIEC mice. When comparing treatment effects between C57BL/6 and RANKΔIEC mice, LysoPS-OVA significantly increased the frequency of CD4^+^ Foxp3^−^ Th3 Tregs in C57BL/6 mice compared to RANKΔIEC mice, whereas mice treated with Free OVA or buffer had a similar frequency of Th3 Treg between the two models.

In addition, LysoPS-OVA treatment led to a significant increase in the frequency of Tr1 Tregs compared to Free OVA and buffer groups, whereas this observation was absent in RANKΔIEC mice (Figure 6C). When comparing treatment effects between C57BL/6 and RANKΔIEC mice, our data showed that LysoPS-OVA induced significantly higher frequencies of Tr1 Tregs in C57BL/6 mice compared to RANKΔIEC mice. These differences were not observed in buffer or Free OVA groups, suggesting that Tr1 Tregs may also be involved in LysoPS-mediated tolerance and that the absence of intestinal M-cells suppressed the generation of Tr1 Tregs.

Foxp3^+^ Treg is a potent regulatory T cell that contributes to immune tolerance. However, our data did not observe significant differences in the frequency of activated Foxp3^+^ Tregs in the spleen of C57BL/6 or RANKΔIEC mice across treatment groups, suggesting Foxp3^+^ Tregs play a minor role in the LysoPS-OVA mediated tolerance responses in the spleen (Supplementary Figure S4B). Interestingly, LysoPS-OVA treatment showed a trend of increased frequencies of Foxp3^+^ Tregs in the bone marrow of C57BL/6 mice, while their frequency was significantly reduced in RANKΔIEC mice (Supplementary Figure S4C). When comparing treatment effects between C57BL/6 and RANKΔIEC mice, we found that both Free OVA and LysoPS-OVA treatment significantly decreased the frequency of activated Foxp3^+^ Tregs in RANKΔIEC mice compared to C57BL/6. These findings suggest that LysoPS-OVA promotes the generation or recruitment of activated Foxp3^+^ Tregs in the bone marrow, potentially contributing to a tolerogenic environment that regulates B cell activation and reduces antibody production.

## 4. Discussion

Previous studies have shown that the structure of PS influences oral tolerogenic potential. Specifically, LysoPS, but not double-chain PS, induces antigen-specific oral tolerance to Factor VIII and GAA in relevant murine models [9]. This observation suggests that the structure of PS influences tolerogenic potential when given orally, possibly due to limited engagement with intestinal M-cells, which are important for transporting PS nanoparticles for oral tolerance. The structure of PS impacts the biophysical characteristics of PS nanoparticles, particularly the PS surface exposure, which is critical for immunological tolerance. LysoPS is a mono-acyl chain phospholipid with a cone-shaped structure, whereas double-chain PS has a cylindrical structure. Incorporation of LysoPS into lipid nanoparticles increases the surface curvature of the vesicles, allowing more LysoPS to partition onto the nanoparticle surface, leading to lipid exposure that is not feasible with double-chain PS [9,28]. Furthermore, previous studies have shown that both LysoPS and double-chain PS have similar biophysical properties (size, TEM morphology, and OVA association efficiency) but differ in PS surface exposure, which impacts downstream tolerogenic potential [9,27,28,29]. This suggests that the difference in immune responses is due to a biological process rather than nanoparticle variation.

Intestinal M-cells are specialized epithelial cells located within the follicle-associated epithelium (FAE) of PPs in the small intestine [12]. They play a central role in maintaining mucosal immune homeostasis by actively sampling luminal antigens and delivering them to the underlying immune inductive sites, particularly the subepithelial dome, where they interact with APCs [30]. M-cells can translocate a broad range of antigens- including dietary proteins, commensal and pathogenic microbes, and particulate antigens such as lipid nanoparticles [12]. Once antigens are translocated to the subepithelial dome, they are captured by resident APC, such as CD103^+^ dendritic cells, which play an important role in oral tolerance. Upon antigen exposure, these DCs upregulate CCR7 expression and migrate to the mesenteric lymph nodes (MLNs) for T and B cell priming [31].

In this study, we investigated the role of intestinal M-cells in LysoPS-mediated oral tolerance. OVA was used as a model antigen, as it has been widely used to study the mechanisms of immune responses [32]. Our data showed that the structure of PS affects nanoparticle uptake by intestinal M-cells. LysoPS nanoparticles, which have higher PS surface exposure than double-chain PS nanoparticles, showed increased transport by intestinal M-cells compared to double-chain PS nanoparticles. These findings suggest that PS exposure can modulate the interaction between nanoparticles and M-cells, thereby affecting nanoparticle uptake and subsequent tolerance induction. One possible explanation is the expression of PS receptors on intestinal M-cells. Studies have shown that M-cells express several PS-binding receptors, such as SR-B1 [33,34], Clusterin [35], and Annexin V [36]. These receptors facilitate the binding of PS and promote internalization of PS nanoparticles by intestinal M-cells. Because LysoPS nanoparticles display increased phosphatidylserine (PS) exposure on their surface, they may interact more efficiently with PS-recognition receptors on M-cells, thereby facilitating binding and transcytosis across the follicle-associated epithelium. M-cells are specialized for sampling particulate antigens through receptor-mediated endocytosis and transcytosis. Previous studies have shown that M-cells express multiple surface molecules, including GP2 and cellular prion protein (PrPᶜ), which facilitate uptake of microbiota-derived components and membrane ligands [36]. In addition to receptor-mediated uptake, M-cells can internalize particulate materials via phagocytosis-like mechanisms. Thus, enhanced PS exposure may promote interactions with these uptake pathways and increase LysoPS nanoparticle internalization. However, the specific receptors involved in the recognition of LysoPS nanoparticles by intestinal M-cells remain unclear.

To evaluate the role of intestinal M-cells in oral tolerance, the OVA-specific IgG antibody titers were assessed and compared between animal models across treatment groups. Our data show that LysoPS-OVA suppressed the OVA-specific IgG production compared to Free OVA in healthy mice. This is consistent with our previous findings demonstrating that LysoPS suppressed the development of antigen-specific antibody responses [9]. However, this suppressive effect is reversed in RANKΔIEC mice, which generated higher OVA-specific IgG titers than C57BL/6 mice despite receiving the same LysoPS-OVA treatment. This observation indicates that intestinal M-cells are required for effective LysoPS-mediated oral tolerance and that the absence of nanoparticle transport to APCs prevents the induction of oral tolerance. These findings suggest that intestinal M-cells are required to initiate tolerogenic immune responses mediated by LysoPS.

TGF-β is an immunosuppressive cytokine that can suppress and regulate immune responses through TGF-β signaling. Studies have shown that the TGF-β cytokine inhibits B cell proliferation and immunoglobulin secretion [37], and promotes Foxp3^+^ Treg differentiation [38]. To assess the role of TGF-β in LysoPS-mediated tolerance, plasma levels of TGF-β1 were measured at week 7, twelve hours following subcutaneous antigen rechallenge. In C57BL/6 mice, LysoPS-OVA significantly increased the concentration of TGF-β1, consistent with our earlier findings that LysoPS-OVA upregulates TGF-β signaling-associated genes in the MLNs [29]. In contrast, RANKΔIEC mice lacking intestinal M-cells displayed a significant reduction in TGF-β1 levels despite receiving LysoPS-OVA. This supports the notion that the generation of TGF-β contributes to LysoPS-mediated oral tolerance, and the absence of intestinal M-cells limits LysoPS-OVA nanoparticle engagement with APCs, thereby reducing TGF-β secretion. Surprisingly, buffer-treated RANKΔIEC mice exhibit elevated baseline TGF-β1 levels compared to C57BL/6 mice, and these animals were not exposed to the OVA during the pre-treatment period. The elevated baseline TGF-β1 levels in RANKΔIEC mice could be due to the compensatory mechanism in the intestinal M cell knock-out model.

Tregs play a critical role in immune regulation and contribute to oral tolerance. In this study, LysoPS induced the generation of Tregs, particularly Th3 Tregs and Tr1 Tregs- two regulatory T cell subsets known to contribute to oral tolerance [39]. Th3 Tregs, which express Latency-Associated Peptide (LAP) on their cell surface, play a significant role in the release of activated TGF-β [40]. LAP^+^ Tregs are 50-fold more immunosuppressive than conventional FoxP3^+^ Tregs in suppressing effector T cells in patients with colorectal cancer, suggesting that LAP^+^ Tregs are a potent immunoregulatory cell type in mucosal immune tolerance [41]. In this study, LysoPS-OVA increased the frequency of Th3 Tregs, which correlated with an increased concentration of TGF-β1 cytokines, supporting a role for Th3 Tregs in LysoPS-mediated oral tolerance. Meanwhile, Tr1 Tregs, another subset of Tregs characterized by the co-expression of LAG-3 and CD49b but lacking Foxp3 expression, also play a key role in maintaining immune homeostasis. Tr1 Tregs are a distinct subset of Tregs that highly express IL-10 and have been reported to contribute to mucosal tolerance [42]. LysoPS-OVA increased the frequency of Tr1 Tregs in C57BL/6 mice but not in RANKΔIEC mice, again suggesting that Tr1 Tregs contributed to LysoPS-mediated oral tolerance, potentially through Interleukin-10 (IL-10) production. Intriguingly, LysoPS did not induce the expression levels of Foxp3^+^ Tregs in the spleen. These conventional Tregs, which are often associated with tolerance induction [43], were not upregulated following LysoPS-OVA treatment. We speculate that these Tregs might play a role in local tolerance responses, which were not investigated in this study. Moreover, Foxp3^+^ Tregs showed a trend toward upregulation in the bone marrow, and the absence of intestinal M-cells significantly reduced the frequency of Foxp3^+^ Tregs after Free OVA and LysoPS-OVA treatment. This observation suggests that Foxp3^+^ Tregs, potentially T follicular regulatory T cells, modulate B cell differentiation at the precursor stage [44], thereby suppressing B-cell differentiation into antibody-secreting plasma cells, which correlates with the reduction in OVA-specific IgG antibodies after LysoPS treatment.

B cell activation leads to plasma cell differentiation, which subsequently drives antibody production. In this study, our data showed that LysoPS-OVA suppressed the frequency of plasma cells in both the spleen and bone marrow, supporting the observation that LysoPS-OVA reduces OVA-specific IgG antibody titers. We speculate that the reduction in plasma cell frequency observed following LysoPS treatment was mediated, at least in part, by the immunoregulatory cytokine TGF-β1, which can act within the spleen and bone marrow to suppress B-cell activation and survival. In addition, LysoPS-induced oral tolerance may impair T follicular helper (Tfh) cells that contribute to the differentiation of germinal center B cells into plasma cells. As a result, fewer plasmablasts would be generated and migrate to the bone marrow, leading to a lower frequency of plasma cells in the bone marrow. Notably, this effect was reversed in mice lacking intestinal M-cells, indicating that LysoPS suppression of antibody production requires M cell function. However, RANKΔIEC mice that received LysoPS developed higher OVA-specific IgG antibody titers than buffer or Free OVA-treated groups, further suggesting that M-cells might play a role in LysoPS-mediated oral tolerance

Recent studies have demonstrated that conventional type-1 DC (cDC1) play a critical role in oral tolerance, especially in inducing peripheral regulatory T cell (pTreg) and promoting antigen-specific immune tolerance [45]. cDC1 are characterized by the absence of CD11b and the presence of CD8α in lymphoid tissues and/or CD103 in non-lymphoid tissues in mice [46]. Studies demonstrated that a high ratio of cDC1/cDC2 DC retains tolerogenic capacity toward OVA during infection, suggesting that cDC1 CD103^+^ play a key role in oral tolerance [47]. In their studies, coculture experiments of dendritic cells (DCs) with naïve CD4^+^ OT-II T cells revealed that pTreg differentiation progressively declined as the ratio of cDC1 to cDC2 decreased, underscoring the importance of cDC1 in pTreg induction. Similarly, in vivo studies further demonstrated the essential role of cDC1 in oral tolerance. For example, during Strongyloides venezuelensis (SV) infection, the immune response suppresses pTreg induction and promotes the expansion of cDC2 cells [47]. Under these conditions, wild-type mice failed to induce pTregs, whereas cDC2-deficient bone marrow chimeras (Zeb2 enhancer triple-mutant) maintained a near-normal cDC1:cDC2 ratio, restored pTreg induction, reduced OVA-specific IgG1 and IgE production, and improved survival in an OVA-induced food allergy mouse model. In addition, our previous single-cell RNA sequencing analysis also highlighted the role of cDC1 in mediating oral tolerance in the MLN [29]. We identified the cluster of cDC1 in the MLN and found that cDC1 also contributed to the outgoing TGF-β signaling to naïve B cells and subsets of T cells, underscoring their tolerance role in LysoPS-mediated tolerance. Therefore, we speculate that LysoPS altered the ratio of cDC1 in the MLN to mediate oral tolerance but certainly require further investigation.

**Figure 7:**
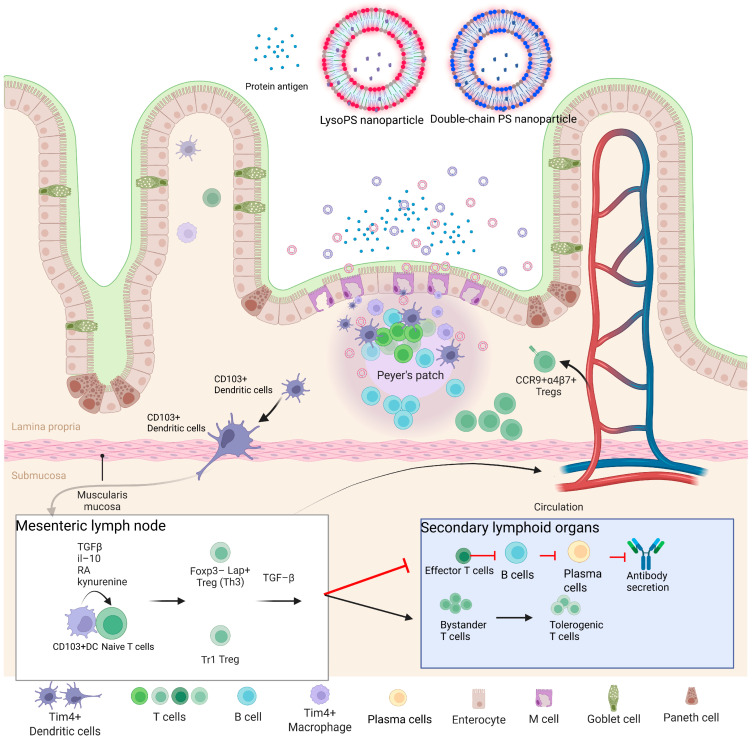
Suggested LysoPS-mediated oral tolerance pathway.

The structure of PS alters the biophysical properties of the PS nanoparticle, particularly PS exposure on the surface, which in turn affects oral tolerance. In this study, we found that intestinal M-cells are critical for LysoPS mediated oral tolerance. The impact of LysoPS mediated oral tolerance is summarized in Figure 7. LysoPS has higher PS exposure than double chain PS, which correlates with increased intestinal M cell uptake, and thereby enhanced nanoparticle transport. This observation is potentially mediated by the engagement of PS nanoparticles with the PS receptors on intestinal M-cells. Once LysoPS nanoparticles are transported into PPs and interact with several APCs, including DCs, macrophages and B cells, antigen presentation occurs and subsequently promotes oral tolerance. However, while DCs, macrophages and B cells are capable of antigen presentation in the PPs [21,48]. Specific APCs that contribute to this process remain unclear. Furthermore, CD103-expressing cDC1 carry LysoPS and migrate into the MLN for antigen presentation to T cells or B cells. In this process, several immunoregulatory mediators such as retinoic acid (RA), kynurenine and immunosuppressive cytokines TGF-β and IL-10, play critical roles in shaping cDC1-driven tolerance responses [45,49]. RA, a vitamin A metabolite, has been shown to prime human DCs to induce gut-homing, IL-10-producing regulatory T cells. Kynurenine, a tryptophan metabolite generated by indoleamine 2,3-dioxygenase 1 (IDO1) in DCs, has been shown to extend their immunoregulatory capacity to the cDC2 via tryptophan metabolism. Through these mechanisms, DC-mediated antigen presentation to T and B cells promotes downstream tolerance, including suppression of B cell activation and the induction of regulatory T cells in the bone marrow and secondary lymphoid tissues, likely through TGF-dependent signaling. Meanwhile, induced regulatory T cells upregulate CCR9 and integrin α4β7 as gut-homing receptors and migrate into gastrointestinal tracts for local immune suppression or tolerization [50].

## 5. Conclusions

Intestinal M-cells play a critical role in PS-mediated oral tolerance. We show here that PS nanoparticles with higher PS exposure correlate with higher uptake by intestinal M-cells, which leads to stronger tolerogenic potential. Following M cell uptake, the downstream mechanism leads to the secretion of the immunosuppressive cytokine TGF-β, which potentially drives the differentiation of Th3 Tregs and promotes a tolerogenic environment. Upregulation of Th3 Tregs also inversely correlates with the suppression of antibody-secreting plasma cells and antibody titers, suggesting that Th3 Tregs may be a biomarker of LysoPS-mediated oral tolerance.

## Data Availability

The original contributions presented in this study are included in the article/Supplementary Materials. Further inquiries can be directed to the corresponding author.

## Supplementary Materials

The following supporting information can be downloaded at: https://www.mdpi.com/article/10.3390/nano16070412/s1, Figure S1. Ligated gut loop displayed that Peyer’s patches contain more LysoPS-OVA nanoparticles. Figure S2. Interaction of PS nanoparticles with immune cells in MLN. Figure S3. Characterization and validation of the in vitro intestinal M cell model. Figure S4. Absence of M-cells reduced activated Foxp3-expressing T cells in the bone marrow.
