# Supplementary material for: Intestinal Microfold Cells Play a Critical Role in the Uptake and Oral Tolerance Mediated by Lysophosphatidylserine-Containing Lipidic Nanoparticles
Source: Nanomaterials (Basel). 2026 Mar 29;16(7):412. doi: 10.3390/nano16070412 (PMC13074963; doi:10.3390/nano16070412)
Supplement: Supplementary file 1 [file nanomaterials-16-00412-s001.zip › nanomaterials-4177818-supplementary.pdf]

Supplementary Materials

A

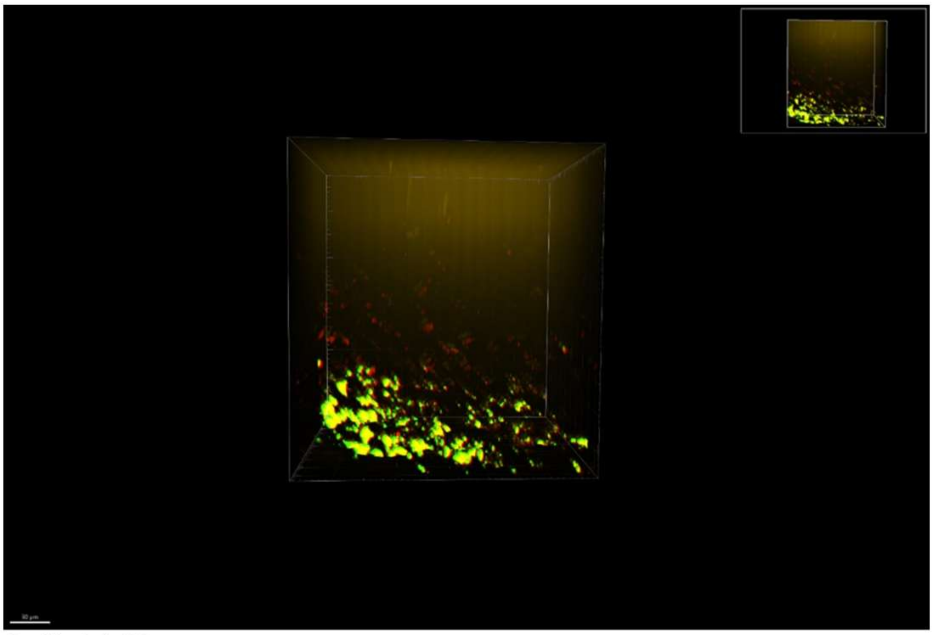

Double-chain PS

B

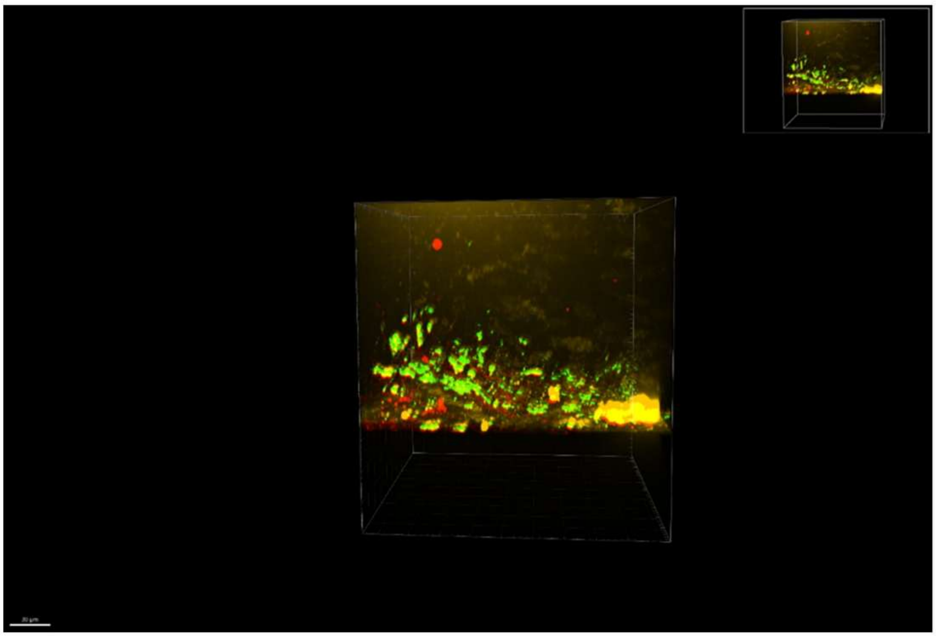

Single-chain PS

Supplementary Figure S1. Ligated gut loop displayed Peyer's patches contain more LysoPS-OVA nanoparticle. Confocal fluorescent images showed the fluorescence of PS nanoparticles (Yellow), OVA protein (Red) and GP-2 markers (Green) in the section of PPs. Images were restructured in 3D images. Scale bar = 20 µm.

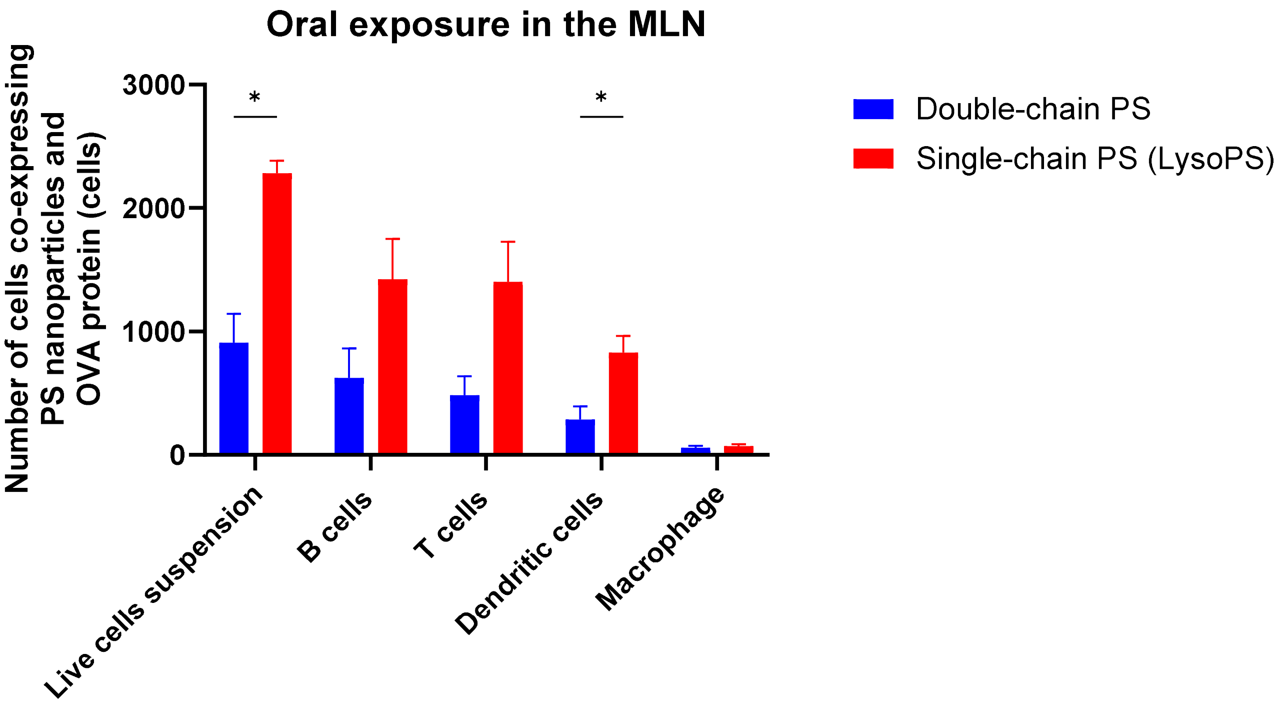

Supplementary Figure S2. Interaction of PS nanoparticles with immune cells in MLN. Fluorescently labeled PS nanoparticles containing fluorescently labeled OVA protein were administered orally to animals. Three hours post-administration, MLNs were collected, digested, and stained to assess co-expression of PS nanoparticles and OVA protein in single-cell suspensions, as well as their distribution among B cells ($B220^{+}$), T cells ($CD3^{+}$), dendritic cells ($CD11c^{+}$), and macrophages ($F4/80^{+}$) ($N = 3$). Error bars represent mean ± SEM. The number of cells co-expressing PS and OVA was quantified using fluorescently labeled counting beads, and dual-positive events were measured by flow cytometry. Statistical analysis was performed using multiple unpaired t-tests without correction for multiple comparisons; significance is indicated as $p < 0.05$.

A

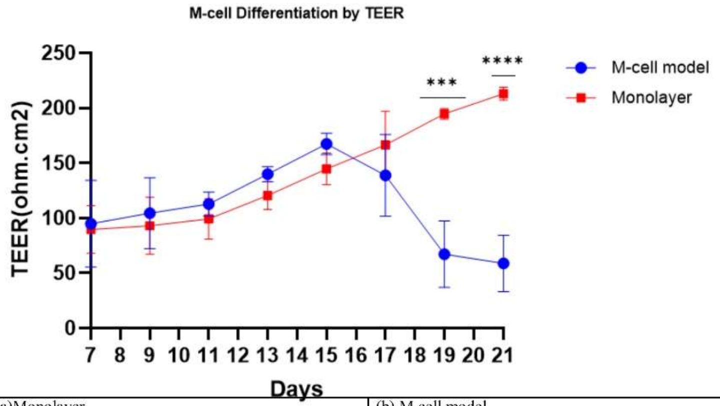

B

	(a)Monolayer	(b) M cell model
GP-2	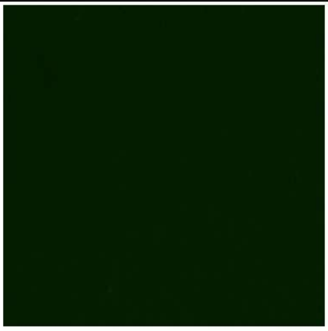	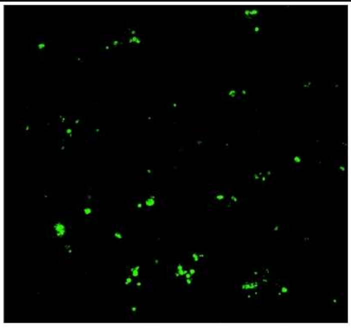
Nuclei	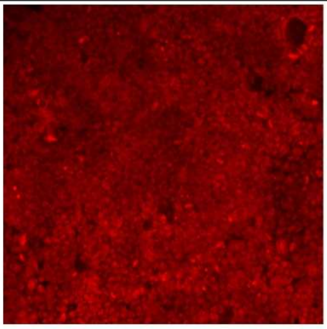	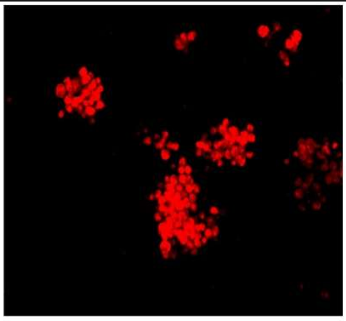
Merged	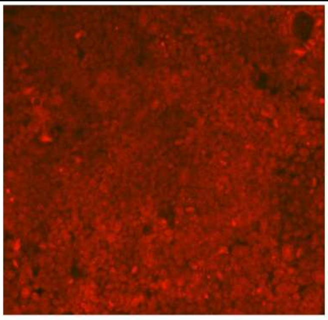	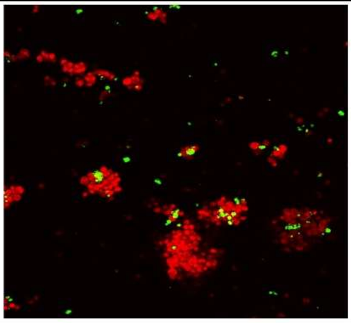

Supplementary Figure S3. Characterization and validation of the in vitro intestinal M-cell model.

(A) Transepithelial electrical resistance (TEER) measurements were used to monitor epithelial barrier integrity and M-cell differentiation over time. Caco-2 monolayers were cultured either alone (monolayer control) or co-cultured with Raji B cells to induce M-cell differentiation. TEER values were recorded from day 7 to day 21 post-seeding. The monolayer group showed a progressive increase in TEER, indicating intact epithelial barrier formation. In contrast, the M-cell co-culture model demonstrated a significant reduction in TEER at later time points, consistent with increased epithelial permeability associated with M-cell differentiation. Data are presented as mean ± SD (n = 3). Statistical significance was determined using one-way ANOVA where ***$p < 0.001$ and ****$p < 0.0001$. Results are plotted as the average of three readings. (B) Immunofluorescence imaging confirming M-cell differentiation using GP-2 as an M-cell marker. Representative images show GP-2 staining (green), TO-PRO3 nuclear staining (red), and merged channels in monolayer and M-cell co-culture conditions. Monolayer controls exhibited minimal GP-2 expression, whereas the M-cell co-culture model showed increased GP-2-positive cells, confirming successful differentiation of intestinal M-like cells. Images were processed using the FIJI/ImageJ software. Scale - 500 µm.

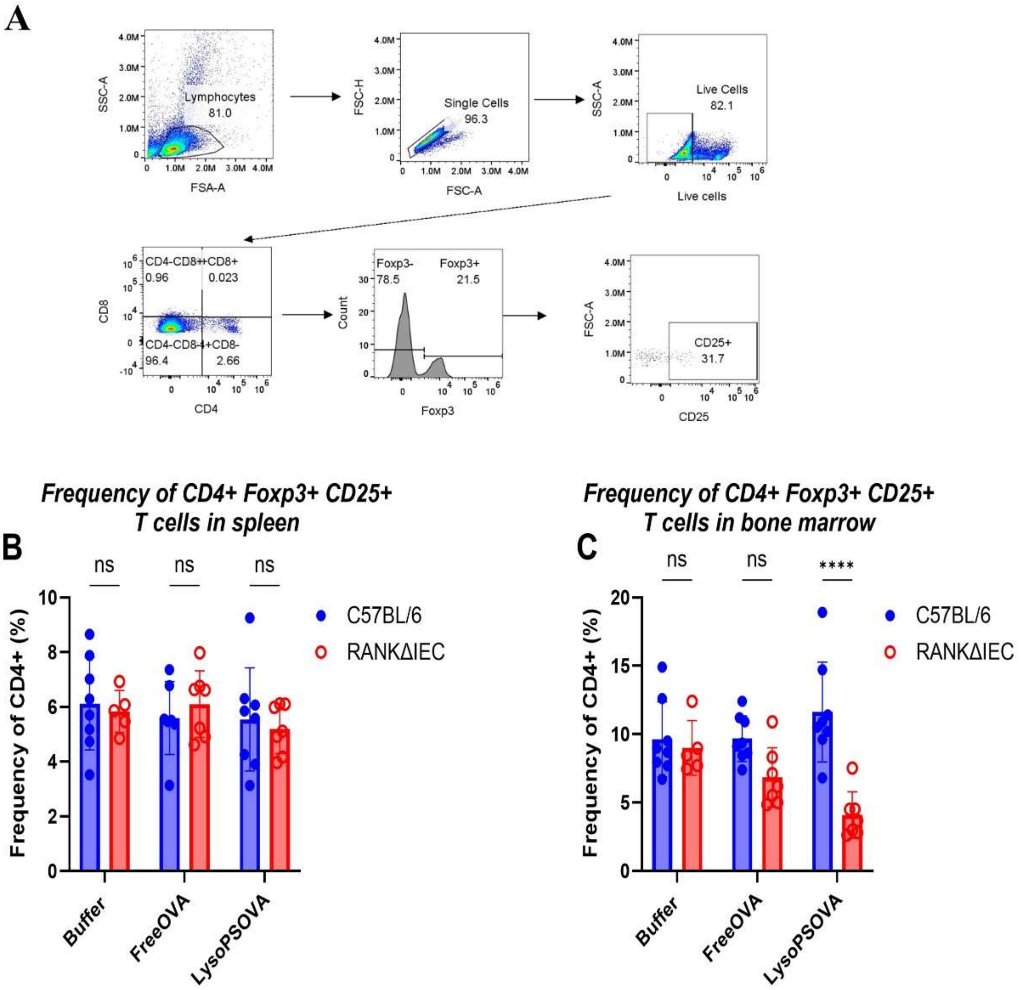

Supplementary Figure S4. Absence of M cells reduced activated Foxp3 expressing T cells in the bone marrow. Frequency of CD4$^+$Foxp3$^+$CD25$^+$ activated regulatory T cells (Foxp3 Tregs) in the spleen and bone marrow. **A)** Gating strategy for Foxp3 Tregs in the spleen and bone marrow. **B)** The frequency of Foxp3+ Tregs across treatment groups in C57BL/6 mice and RANKΔIEC mice in the spleen. **C)** The frequency of Foxp3+ Tregs across treatment groups in C57BL/6 mice and RANKΔIEC mice in the bone marrow. Statistical significance was assessed using two-way ANOVA to compare differences between C57BL/6 and RANKΔIEC with Bonferroni multiple comparison. $p < 0.05$; *$p < 0.01$; **$p < 0.001$; ns, not significant.
